# The relative plasma availabilities of ivermectin in reindeer (*Rangifer tarandus tarandus*) following subcutaneous and two different oral formulation applications

**DOI:** 10.1186/s13028-014-0076-9

**Published:** 2014-11-25

**Authors:** Antti Oksanen, Kjetil Åsbakk, Marja Raekallio, Mauri Nieminen

**Affiliations:** Finnish Food Safety Authority Evira, Production Animal and Wildlife Health Research Unit (FINPAR), Elektroniikkatie 3, FI-90590 Oulu, Finland; Department of Arctic and Marine Biology, Research Group of Arctic Infection Biology, University of Tromsø, P.O. Box 6050, Langnes, NO-9037 Tromsø, Norway; Faculty of Veterinary Medicine, Department of Equine and Small Animal Medicine, Pharmacology and Toxicology Section, University of Helsinki, P.O. Box 57, FI-00014 Helsinki, Finland; Finnish Game and Fisheries Research Institute, Reindeer Research Station, FI-99910 Kaamanen, Finland

**Keywords:** Reindeer, Ivermectin, Injection, Drench, Paste, AUC

## Abstract

**Background:**

Overwintering (breeding) reindeer (*Rangifer tarandus tarandus*) are commonly treated with ivermectin against parasitic infestations once yearly in autumn-winter roundups. The only preparations registered to reindeer are those for subcutaneous injection. However, also oral extra-label ivermectin administration is used. Twenty-six, 8-month-old reindeer calves were randomly allocated into three groups. Group 1 (n = 9) received oral ivermectin mixture (Ivomec® vet mixt. 0.8 mg/ml, oral ovine liquid drench formulation), Group 2 (n = 9) oral ivermectin paste (Ivomec® vet 18.7 mg/g equine paste), and Group 3 (n = 8) subcutaneous injection of ivermectin (Ivomec® 10 mg/ml vet inj.), each group at a dose of 200 μg/kg body weight. Blood samples were collected at treatment and at days 1, 2, 3, 6, 9 and 16 post treatment. Plasma concentrations of ivermectin were determined by high-pressure liquid chromatography (HPLC) with fluorescence detection.

**Results:**

The peak plasma concentration (C_max_) was reached by 2 days after each treatment. The C_max_ and Area Under Curve (AUC) differed significantly between the groups: C_max_ was 30.2 ± 3.9, 14.9 ± 5.7 and 63.1 ± 13.1 ng/ml, and AUC_∞_ was 2881 ± 462, 1299 ± 342 and 6718 ± 1620 ng*h/ml for groups 1, 2 and 3, respectively (mean ± standard deviation).

**Conclusions:**

The differences in plasma concentrations of ivermectin are concomitant with earlier observed differences in antiparasitic efficacy, which discounts the use of the equine paste in reindeer in favour of the oral ovine liquid drench formulation, or preferably, the reindeer-registered subcutaneous injection formulation.

## Background

Since the launch at the production animal market, ivermectin has been widely used to treat different endoparasite and ectoparasite (nematodes and arthropods) infections in the main production animal species [1], and also in “minor” ruminant species such as goats, deer, buffaloes, antelopes, and also camelids. In a review of extra-label use in minor species [2], it was concluded that there is a high inter-species variability in ivermectin pharmacokinetics and efficacy and a need for extended pharmacokinetic data in various animal species to avoid misuse of dose rates extrapolated from other species.

Ivermectin has been used to treat both arthropod and nematode parasites of reindeer (*Rangifer tarandus tarandus*) since the early 1980s [3,4] with as many as 80% of the overwintering reindeer in Finland treated once yearly [5]. The only ivermectin preparation registered for administration to reindeer in any Fennoscandian country is injectable (e.g. Ivomec® 10 mg/ml vet inj., Merial), at 200 μg/kg subcutaneously (s.c.). The equine oral paste (Ivomec® vet 18.7 mg/g paste, Merial) is not intended by the manufacturer for use in ruminants. It was, however, adopted to the treatment of reindeer in Finland after reindeer herders had seen that administration at 200 μg/kg had high efficacy against reindeer warbles (*Hypoderma tarandi*) and throat bots (*Cephenemyia trompe*), which effect was later demonstrated experimentally [6]. The oral ovine liquid drench formulation (Ivomec® vet mixt., 0.8 mg/ml, Merial) is occasionally used on reindeer in Norway (Aamot, Herdis G., pers. comm., 2013). This study compares the plasma concentrations of ivermectin in reindeer after administration of the oral ovine liquid drench and equine paste formulations, and the subcutaneous injection, and discusses the consequences of the observed differences to antiparasitic effect and resistance.

## Material and methods

Twenty-six (13 females and 13 males) individually ear-tagged 8-month-old reindeer calves of the Kaamanen Experimental Reindeer Herd, Kaamanen, Finnish Lapland, were corralled at the Finnish Game and Fisheries Research Institute’s Reindeer Research Station animal experiment facilities and fed lichen and commercial reindeer feed pellets (Poron-Herkku, Rehu-Raisio, Finland) ad lib. The animals were weighed (median body weight 46 kg, range 42 to 53 kg) and allocated by sex and weight into three treatment groups: 1) ovine Ivomec® vet mixt. 0.8 mg/ml, n = 9; 2) equine Ivomec® vet 18.7 mg/g paste, n = 9; and 3) Ivomec® 10 mg/ml vet inj., n = 8. The treatments were performed after pelleted fodder had been added to their feed troughs in the morning. All animals were dosed at 200 μg/kg body weight. Those in Groups 1 and 2 were treated by emptying an individually filled syringe on the base of the tongue, 0.25 ml/kg and 10.7 mg/kg, respectively. Subcutaneous injections (Group 3) were given in front of the left shoulder (lateral midline of the neck) at 0.02 ml/kg. Blood samples were collected into evacuated sodium heparinised 10 ml tubes (Venoject) immediately prior to treatment (day 0) and at the same time of day on days 1, 2, 3, 6, 9 and 16 post treatment. Plasma was separated by centrifugation, stored at -20°C, and only thawed once for analysis.

The experiment was performed with the permission from Finnish Game and Fisheries Research Institute’s Animal Care Committee.

Ivermectin concentration in plasma was determined by high-pressure liquid chromatography (HPLC) with fluorescence detection following a modification of the method described for ivermectin in reindeer faeces [7,8]. The modifications included that the 1.0 ml plasma sample was supplemented with 10 ng of abamectin internal standard, mixed with 5 ml of 30% acetone, and the iso-octane extraction step was performed with 5 ml portions of iso-octane. A single calibration line based on the least-squares method was used for the quantification of ivermectin. Concentrations were calculated according to the equation:

$$ {C}_s = \left(\frac{R_{f1}{H}_i}{H_a}+{R}_{f2}\right)\frac{W_a}{W_s}{D}_f $$

where Cs is the plasma concentration (ng/ml); Rf1 and Rf2 are the response factors representing the calibration line used, 1.1777 and 0, respectively; Hi and Ha are the HPLC software-generated heights of the ivermectin B1a and abamectin Bla peaks, respectively; Wa is the internal standard weight (ng); Ws, the sample volume (ml); Df, the dilution factor for sample injected. For recovery calculation, 1.0 ml plasma portions from untreated animals fortified with concentrations of ivermectin ranging from 1 to 100 ng/ml were analysed, and recovery was calculated according to equation published before [7].

**Figure 1:**
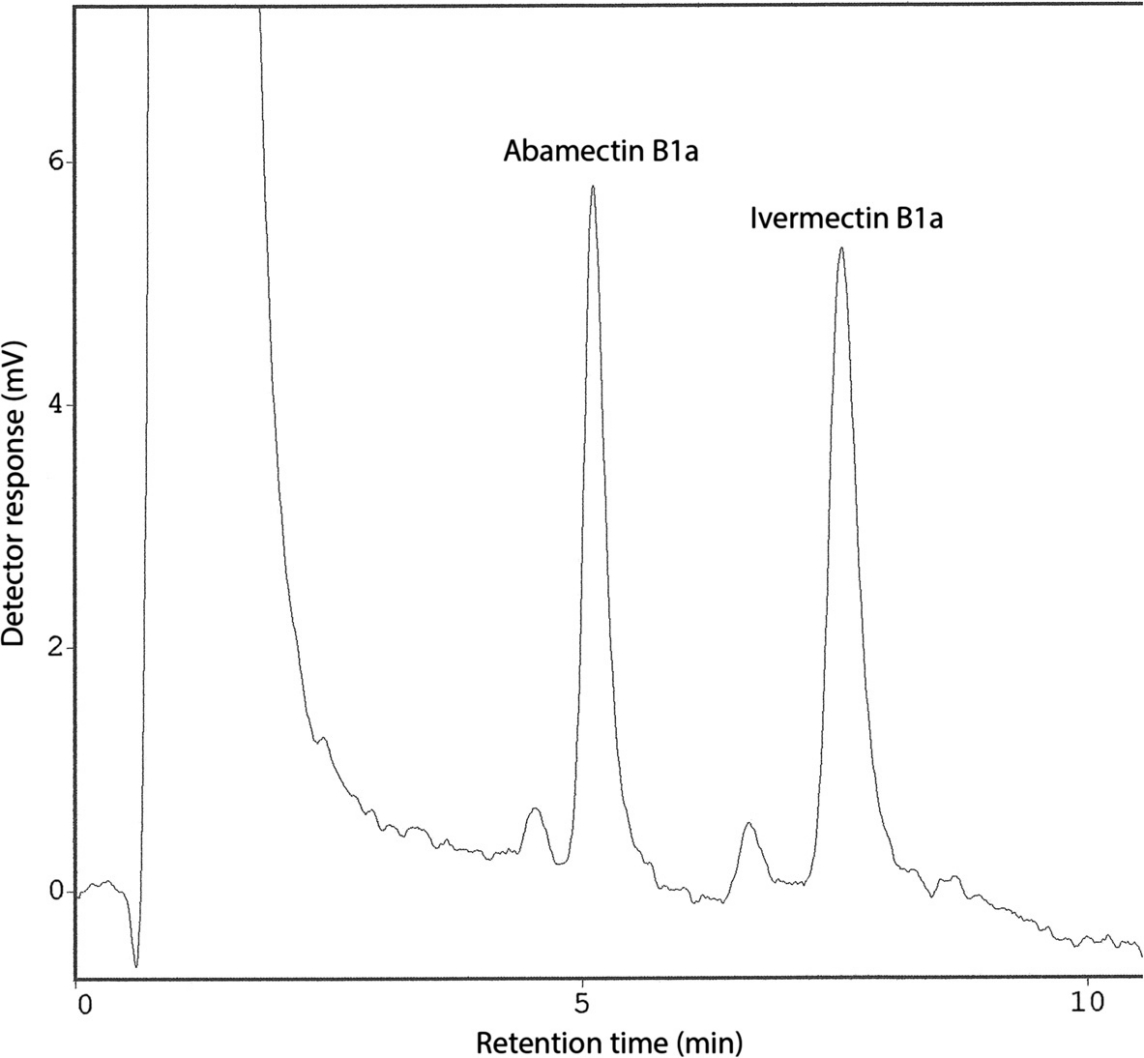
**An example HPLC chromatogram for an actual plasma sample from a reindeer treated with ivermectin by oral administration of equine paste (Group 2) at the dose of 200 μg/kg.** Sample from day 2 post treatment, concentration determined to 9 ng/ml. HPLC detector attenuation: 3. Each plasma sample for analysis (1.0 ml) was supplemented with 10 ng abamectin (internal standard) before extraction and sample preparation. The plasma concentration calculation was based on the HPLC software-generated heights of the ivermectin B1a and abamectin Bla peaks using the equation given above.

The plasma ivermectin concentration-time profiles were examined using WinNonLin 6.3 (Statistical Consulting Inc. Pharsight Corporation, Cary, NC, USA) software with non-compartmental analysis, linear trapezoidal linear/log interpolation, uniform weighting. Appropriate basic pharmacokinetic parameters, mean and standard deviation, were generated. For calculation, the first ivermectin concentration from an animal below the level that could be recovered and determined (1 ng/ml) was replaced with 0.5 ng/ml. The relative plasma availability of ivermectin (F_rel_) was approximated by using the mean AUCs of each group and calculating (AUC_oral_/AUC_sc_)*100%, where s.c. route was used as a reference. Differences in maximum concentration (C_max_) and Area Under the plasma ivermectin concentration-time Curve (AUC_∞_) between treatments were analyzed with analysis of variances by JMP statistical software (ver. 11, SAS Institute Inc., www.jmp.com). The null hypothesis was rejected at an α-level of 0.05.

## Results

Chromatography of 1.0 ml plasma samples from an untreated reindeer fortified with various amounts of ivermectin demonstrated that fortification levels down to 1 ng/ml could easily be detected and measured by HPLC. Retention times for the Bla components of abamectin and ivermectin were approximately 5.1 and 7.5 min, respectively, with the Blb components eluting a little less than 1 min earlier (Figure 1). The individual peaks were well resolved at all fortification levels, and no extraneous peaks interfered with the Bla components, as demonstrated by analysis of non-fortified plasma from untreated animals. The relative recovery rate ranged from ~99 to ~116% for the whole concentration range (1-100 ng/ml), and reproducibility was good (standard deviation less than 5.2% for concentration determined for 5 identically prepared samples).

One animal of Group 3 gave analysis results showing complete absence of ivermectin in all samples and was excluded as an untreated animal. In addition, of the 175 samples of other animals, 17 were missing from analysis for various reasons.

**Figure 2:**
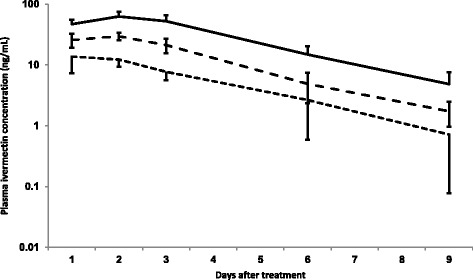
**Mean (** **+** **standard deviation) plasma ivermectin concentration (ng/ml) in three groups of reindeer treated with ivermectin (x-axis: days after treatment),** ***1) broken line,*** **Ivomec** **®** **vet mixt. 0.8 mg/ml orally n = 9;** ***2) dotted line,*** **Ivomec** **®** **vet 18.7 mg/g paste orally n = 9** ***; 3) solid line,*** **Ivomec** **®** **10 mg/ml vet inj. subcutaneously n = 8** ***.*** All treatments at a dose of 200 μg/kg.

Mean plasma ivermectin concentrations by time post treatment for each group are shown in Figure 2. The maximum measured ivermectin concentration (C_max_) was reached by day 2 post treatment (T_max_). One animal in Group 2 exhibited an exceptionally high plasma ivermectin concentration on day 1 (29 ng/ml, compared to the mean of 11.8 ng/ml for the other eight of the group) and caused the mean concentration in that group in Figure 2 to peak on day 1 (mean = 13.7, day 2 mean = 12.2 ng/ml). The C_max_ and AUC differed significantly between the groups (Table 1), and the mean C_max_ and AUC were significantly lower for Group 2 compared to Groups 1 and 3, and significantly higher for Group 3 compared to Groups 1 and 2 (one way ANOVA, Tukey test, *P* < 0.007). No ivermectin could be detected on day 16 post treatment in any animal indicating that the plasma concentrations were below the detection limit (1 ng/ml). The approximated F_rel_ were 43% and 19% for the oral mixture and paste, respectively.

**Table 1:** **Basic pharmacokinetic parameters of ivermectin administered orally (as mixture or paste), or subcutaneously to reindeer calves at 200 μg/kg, mean and standard deviation**

**Parameter**	**Group 1, mixture**	**Group 2, paste**	**Group 3, injection**
T_max_ (days)	2	1	2
C_max_ (ng/ml)	30.2 ± 3.9^a^	14.9 ± 5.7^b^	63.1 ± 13.1^c^
AUC_∞_ (ng*h/ml)	2881 ± 462^a^	1299 ± 342^b^	6718 ± 1620^c^
F_rel_	0.43	0.19	1

Means of the groups marked with different letters (a, b, c) differ significantly (*P* < 0.05).

## Discussion

The maximum ivermectin plasma concentrations measured in the subcutaneously injected reindeer were higher in this study than in a previous one with similar animals [9]. In that earlier experiment, no sampling was done on day 2 after treatment, but only on days 1 and 3, and thus C_max_ was probably missed. Otherwise, the results from these two experiments were similar, suggesting the superior plasma availability of ivermectin after subcutaneous injection compared to oral administration. Maybe C_max_ were not seen in the current study either, because they may be anywhere between days 1 and 2, or between days 2 and 3. The T_max_ of ivermectin in reindeer is in any case longer than in some other cervid species where it has been less than 1 day (see below) but shorter than or similar to cattle, sheep and goats (reviewed in [2]). The C_max_ following subcutaneous administration at 200 μg/kg was considerably higher in our study than observed in cattle [10-12].

It is most interesting to compare the findings with results from other cervid species. After s.c. injection of ivermectin in red deer (*Cervus elaphus*) at 200 μg/kg, C_max_ was only 15.8 ng/ml [13], about one fourth of that measured in the current study. In red deer the T_max_ was 20 hours. Similar values were obtained in another study in red deer [14]. In white-tailed deer (*Odocoileus virginianus*) sampled days 3, 7, 14, and 21 post injection, C_max_ was observed in the first sampling, on day 3 post injection; the serum concentration was about 30 ng/ml, almost double the C_max_ in red deer [15]. The elimination to non-detectable serum concentration (<2 ppb ~ 2 ng/ml) lasted not more than 21 days after injection [15]. However, these findings are not fully comparable, as the sampling intervals differed between studies and the peak concentrations may not have been measured due to infrequent sampling. In fallow deer (*Cervus dama dama*), injectable abamectin (200 μg/kg, s.c.), chemically a closely related and pharmacologically rather similar drug, produced a C_max_ of 120 ng/ml at 19 h post injection [16]. In spite of the similarities, ivermectin is regarded as the least lipophilic of the macrocyclic lactone antiparasitics [17], which probably causes differences between the pharmacokinetics of these two compounds. As stated [2], erroneous dosages may emerge when extrapolating recommended treatments from one animal species to another. This appears very clearly evident also within the Cervidae family.

In the present study, the elimination half-time and mean residence time could not be calculated accurately, as the slow absorption was probably a limiting factor for the elimination causing the so called flip-flop pharmacokinetics. Thus the main, and clinically most important, differences between the groups were in C_max_ and AUC. Plasma concentration, or specifically AUC, of a macrocyclic lactone in a given animal species has been suggested to indicate the level of antiparasitic efficacy [18]. It was suggested that there may be a minimum plasma drug concentration (between 0.5 and 1 ng/ml [19] for optimal anthelmintic activity against susceptible gastrointestinal or lung nematodes. However, the existence of such a minimum effective concentration has not been confirmed. In any case, the efficacy concentration and duration requirements are affected by physiological and biochemical differences between both host and parasite species and strains.

Even though more sensitive analytical methods do exist [20], we consider our method appropriate for the focus of the present work, the comparison between the practical outcomes of the three administration regimes in question, particularly when laboratory cost-effectiveness and labour-intensity also are taken into account. An important problem with the relatively low analytical sensitivity of the method is that it was not possible to detect the prolonged low concentrations which may be important in anthelmintic resistance build-up in nematode populations. Furthermore, the elimination parameters could not be calculated.

The efficacy of ivermectin formulations against gastrointestinal trichostrongylid nematodes in reindeer has been assessed in several previous studies based on the reduction in faecal egg output in the Kaamanen Herd. The s.c. injection treatment reduced the faecal egg count mean (based on several repeated samplings of individual animals 2 to 6 months post treatment) by 80-95% [21,22]; the equine paste by 39% [21], and the ovine mixture by 62 to 74% [23]. These efficacy figures were consistent both with the C_max_ and AUC differences detected in the present study. Based on C_max_ and AUC comparisons between the different preparations and administrations, the equine ivermectin paste may have similar antiparasitic efficacy, at least against extra-intestinal parasites, to a lower dose of the ovine mixture, or to an even lower dose injected subcutaneously, which low injection dose would naturally be regarded as evident underdosage.

The reason for the obviously better absorption to the circulation of ivermectin from the ovine mixture compared to the equine paste may be in the chemical composition of the base material of the products, but one possible explanation is in the consistence and volume of the drench. The equine paste dose containing 200 μg/kg of ivermectin and given to a 50 kg reindeer calf is 0.535 g of viscous paste, while that of the ovine mixture is 12.5 ml of liquid. If some of the mixture bypasses the rumen to end up directly in the abomasum, the absorption to the body might be greatly enhanced. In an early study in sheep, the bioavailability of intra-abomasally administered ivermectin was 100%, while that of ivermectin administered intraruminally was only 25% [24]. It was suggested that orally administered ivermectin is adsorbed to the ruminal ingesta [24,25], which limits its availability for absorption. Accordingly, also the systemic availability of intraruminally administered doramectin in sheep is low, 35% [26]. In our study, the approximated relative plasma availability was lower for the paste and higher for the mixture in reindeer than these values reported for intraruminal administration in sheep [24].

Ruminal bypass could be explained by the oesophageal groove closure directing the drug mixture directly to the abomasum. This explanation loses some credibility because ruminal bypass was found not to take place in adult reindeer [27]. The 8-month-old reindeer calves in the present study had recently been weaned; the end of lactation takes place typically 24 to 26 weeks after birth [28]. Possibly the animals’ raised head posture during administration anyhow directs ivermectin mixture to the abomasum. It can also be difficult to make sure that the reindeer swallow the small volume of paste. In this study, proper restraint of the animals ensured swallowing.

We are not aware of any reports of macrocyclic lactone resistance in any oestrid fly species, such as reindeer warble and throat bot flies, but resistance in gastrointestinal nematode parasites is a major problem in many areas where e.g. sheep are intensively treated [29,30]. We do not know of any report of ivermectin resistance in any reindeer parasites. However, after two decades of moxidectin pour-on use on a New Zealand deer farm, significant resistance to moxidectin and abamectin in abomasal nematodes was seen [31]. Moxidectin is apparently not used in reindeer, as it was found not to have satisfactory efficacy against the important insect parasites, warbles and throat bots [22]. Pour-on ivermectin preparation was found to have very low absorption into the reindeer body, especially in midwinter [9].

As underdosage is regarded as a driver of resistance [32], it should definitely be avoided. On the other hand, persistent activity of macrocyclic lactones creates situations in which treated animals have prolonged low concentration of the drug in the body, which may allow (re) infection by the fittest of parasite larvae [33]. From that point of view, rapid disappearance of the drug would be preferable, and there oral administrations probably score highest. Ivermectin is mostly excreted unaltered in the faeces [34], and the environmental contamination [8] will be similar in size, but not in time, regardless the application route.

However, systemic plasma availability is probably not essential as a measure of antiparasitic activity; it is the availability of the toxic substance to the parasites that really counts. While plasma concentration virtually certainly determines the availability of ivermectin to parenteral nematodes (such as e.g. *Dictyocaulus eckerti, Setaria tundra* and *Rumenfilaria andersoni*) and warble and throat bot fly larvae, the situation regarding gastrointestinal nematodes is somewhat confusing. In recent studies in sheep and cattle, oral macrocyclic lactones have been shown to have higher efficacy against gastrointestinal nematodes than subcutaneous injection [35,36], even against *Haemonchus contortus*, which is known to feed on blood. One probable explanation to the observed difference in effect against reindeer abomasal nematodes (*Ostertagia gruehneri*) [37] and those of sheep and cattle, is that during the time of treatment, reindeer nematodes mostly reside in the abomasal mucosa as inhibited larvae [37,38], and cannot get in contact with drug in ingesta; thus, ivermectin is only available to them via blood circulation.

## Conclusions

This study has defined the plasma concentrations of ivermectin in reindeer using three formulations, and the results agree well with previously acquired anthelmintic efficacy data. With this information, some directions for the best use of drug can be made. Specifically, if an injection is impractical to administer e.g. due to lack of capable personnell, and ivermectin treatment is still required, the higher relative plasma availability of ivermectin in the mixture formulation, designed to be used in a ruminant species, suggests that it would be a better substitute than the paste designed for a monogastric species.
